# Investigation of monolithic passively mode-locked quantum dot lasers with extremely low repetition frequency

**DOI:** 10.1186/s11671-014-0720-3

**Published:** 2015-01-31

**Authors:** Tianhong Xu, Juncheng Cao, Ivo Montrosset

**Affiliations:** Key Laboratory of Terahertz Solid-State Technology, Shanghai Institute of Microsystem and Information Technology, Chinese Academy of Sciences, Shanghai, 200050 China; Dipartimento di Elettronica e Telecomunicazioni, Politecnico di Torino, Turin, 10129 Italy

**Keywords:** Mode-locked lasers, Quantum dot, Delayed differential equation, Bifurcation, Semiconductor device modeling

## Abstract

The dynamical regimes and performance optimization of quantum dot monolithic passively mode-locked lasers with extremely low repetition rate are investigated using the numerical method. A modified multisection delayed differential equation model is proposed to accomplish simulations of both two-section and three-section passively mode-locked lasers with long cavity. According to the numerical simulations, it is shown that fundamental and harmonic mode-locking regimes can be multistable over a wide current range. These dynamic regimes are studied, and the reasons for their existence are explained. In addition, we demonstrate that fundamental pulses with higher peak power can be achieved when the laser is designed to work in a region with smaller differential gain.

## Background

The quantum dot (QD) semiconductor material for mode-locking (ML) lasers is an intrinsically suitable active-region material for short- and high-power pulse generation, due to its unique properties such as fast gain dynamics, easy gain/absorption saturation, and small linewidth enhancement factor [1]. It has already been experimentally demonstrated that a short periodic pulse sequence can be obtained by passive ML using two-section Fabry-Perot (FP) cavity lasers [2]. Generally, high repetition frequency QD ML lasers (from tens of GHz to hundreds of GHz) and their performance improvement are the focus of many papers (see review paper [1] and references there). However, only few papers study the monolithic QD ML lasers with relatively low repetition frequency and, especially, their dynamical regimes [3,4]. Indeed, in some applications, such as micro-machining and two-photon microscopy, periodic high-power pulse sequences with much lower repetition rate are more desirable.

In this work, the dynamic working regimes of a 2-cm monolithic QD ML laser with a repetition frequency of about 2.4 GHz have been studied utilizing a modified delayed differential equation (DDE) model. Fundamental and harmonic ML regimes have been observed when changing the injection current, and the multistable regime sustains over a large current range. We found that the launch of the harmonic ML at high current should be mainly attributed to the relatively long repetition period of the device and the changes in the gain/absorption dynamics with the current. Based on numerical simulations, we demonstrated a way to push the starting point of the multistable regime to a higher current value, in order to obtain higher output peak power in stable fundamental ML mode.

## Methods

**Figure 1:**
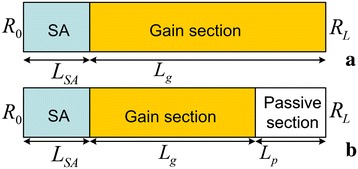
**Schematic diagram of the considered two-section (a) and three-section (b) passively ML FP lasers in this paper.**

Unless otherwise specified, in this paper, we considered a monolithic two-section QD ML laser (as shown in Figure 1a) with active region consisting of fivefold stack of self-assembled InAs QD layers and with a saturable absorber (SA) length *L*_SA_ of 2 mm, gain section length *L*_g_ of 18 mm (the corresponding fundamental repetition frequency is 2.4 GHz), ridge width *W* of 6 μm, high-reflection coating at the SA side facet with reflectivity *R*_0_ = 95%, and the cleaved output facet with reflectivity *R*_*L*_ = 33%. Indeed, this monolithic laser, used in our numerical simulation, has the same waveguide structure parameters to those used in the experimental studies in [3].

In previous publications [5,6], we have proposed a multisection DDE model, fully accounting for the ultra-fast carrier dynamics in the active region and the phase locking of the longitudinal modes in the waveguide of the QD ML lasers (for detailed descriptions of this model, please refer to [5]). However, to perform the investigation of low repetition rate lasers, the previous model has been modified.

Firstly, modal gain description has been changed. In the old DDE model, we assume that the hole occupation probability in the valence band *ρ*^*h*^ equals exactly to the electron occupation probability in corresponding states in the conduction band *ρ*^*e*^, which is the so-called excitonic model. While, in this paper, a quasi-exitonic DDE model is exploited, where we consider independent hole occupation probability in the gain section. *ρ*^*h*^ is assumed to have a fixed value in the gain section, to take into account the fact that, at moderate current density, the quasi-Fermi levels in the valence band are always clamped, due to the small separations between each sub-band in the valance band [7]. Therefore, in this quasi-exitonic DDE model, the modal gain *g*_*i*_ in the gain section is calculated following the equation:

1 $$ {g}_i={\varGamma}_x{\varGamma}_y{g}_{0\_i}\left({\rho}_i^e+{\rho}_i^h-1\right),\;\left(i=\mathrm{G}\mathrm{S},\;\mathrm{E}\mathrm{S}\right) $$

where *g*_0_ is the material gain coefficient, *Γ*_*x*_ = 0.65 and *Γ*_*y*_ = 0.0675 are the confinement factors in the lateral and vertical directions, respectively. We assume that *ρ*^*h*^_*i*_ has constant values of 0.4 and 0.2 for the ground state (GS) and the excited state (ES), respectively [8], and the material gain coefficients of the GS and ES transitions are 585 and 972 cm^−1^, respectively. On the contrary, in the SA, where no current injection is applied and electrons and holes are generated by the photon absorption, *ρ*^*h*^ should be equal to *ρ*^*e*^, so the modal absorption *а*_SA*_i*_ in the SA can be written in the form:

2 $$ {\alpha}_{\mathrm{SA}\_i}={\varGamma}_x{\varGamma}_y{g}_{0\_i}\left(1-2{\rho}_i^e\right),\;\left(i=\mathrm{G}\mathrm{S},\;\mathrm{E}\mathrm{S}\right) $$

According to Equations 1 and 2, we can easily derive that, since *ρ*^*h*^ is fixed to a relatively low value (compared with the high *ρ*^*e*^, which is always larger than 0.8), the maximum modal gain (when *ρ*^*e*^ = 1) is usually smaller than *Γ*_*x*_*Γ*_*y*_*g*_0_, i.e., the maximum modal gain is always smaller than the unsaturated absorption *а*_SA_0_ (when *ρ*^*e*^ = 0). These choices for the gain representation are in good agreement with the experimental observations (see Figure three in [1]), proving the validity of the quasi-exitonic assumption.

Secondly, the gain spectral bandwidth 2*γћ* has been reduced. We have referred to the value used in Vladimirov's paper (see Figure two in [9]), so 2*γћ* = 3.4 meV, which is ten times smaller than the value we used in [6]. Other simulation-related parameters are the same as those in [6].

Thirdly, we have developed a modified multisection DDE model for the simulations of the three-section QD ML laser. In the last part of this work, a three-section QD ML laser has been studied, in which the additional passive section (see Figure 1b) has the same active region with the gain/SA sections but is biased with an appropriate level of current to achieve optical transparency, i.e., there is no active region induced optical amplification or absorption. The simulations of this device have been performed by means of a modified DDE model, in which the passive section has been considered as an additional section that is optically transparent but has the same refractive index and intrinsic waveguide losses as those in the other two sections. This absorber-gain-passive design was previously proposed in [10].

## Results and discussion

### Dynamic regimes of the monolithic two-section ML laser

**Figure 2:**
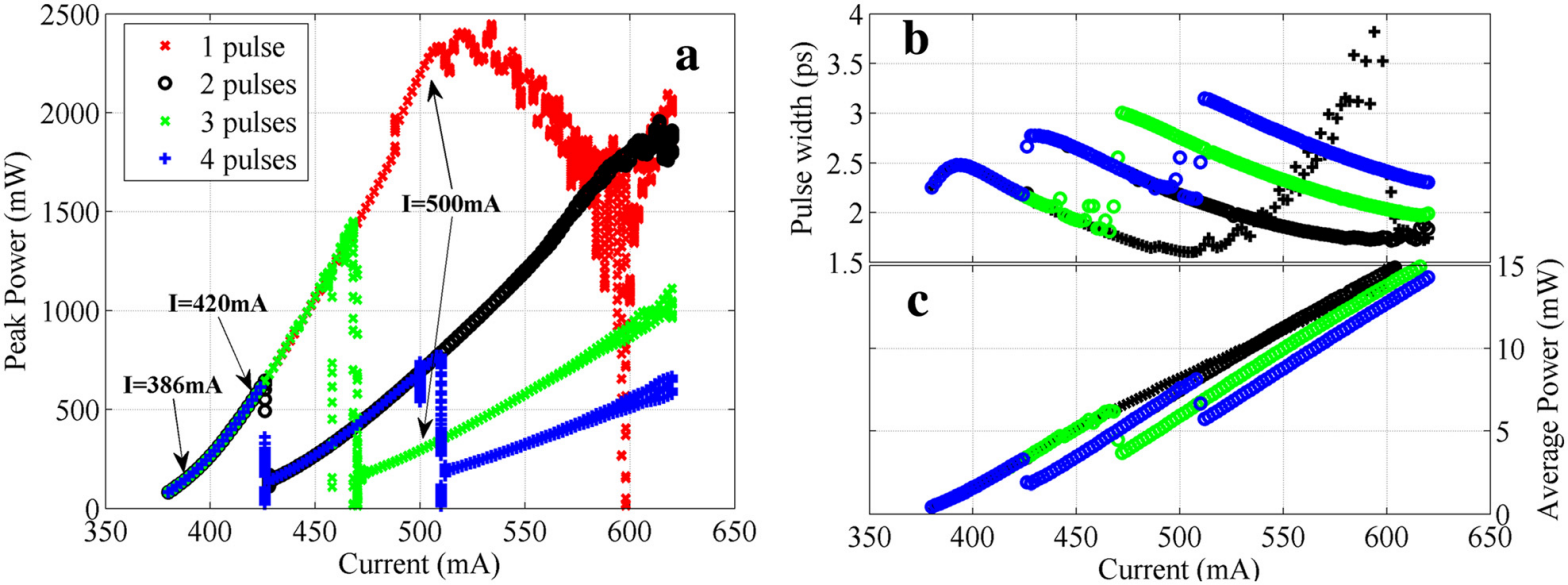
**Bifurcation diagrams illustrating different operation regimes of the 2**-**cm-long two-section ML laser. (a)** Peak power, **(b)** pulse width, and **(c)** average power at the fixed reverse-bias voltage *V* = −2 V and as a function of the injection current for this device are shown. The fundamental ML branch (red cross) and the second (black circle), the third (green cross), and the fourth (blue plus) harmonic ML branches are illustrated. Arrows in (a) identify the working point which will be discussed later in Figure 3.

In this subsection, we analyze the dynamic regimes for the previously described 2-cm-long two-section passively ML laser. In Figure 2, the bifurcation diagrams of the achieved peak power, pulse width, and average power are reported.

Particularly, in Figure 2a, at each current point, local maxima of the output optical power time trace over the last 20 round trips are gathered and plotted, showing the pulse peak power stability/fluctuation in such time interval. This diagram is obtained by fixing the reverse voltage at −2 V and sweeping the injection current in a range from 380 mA (the threshold current) to 620 mA back and forth with a decreasing/increasing step of 2 mA for the harmonic ML/fundamental ML branches, respectively. Considering the second harmonic branch as an example, at the initial current point (620 mA), the device is seeded with a well-defined initial excitation trace, which has 1-mW peak power, 1-ps pulse width, and two equal-spaced Gaussian-shape pulses within one round trip time; for the following current steps, the device is excited using the result of the last simulation with 2 mA higher current. Other branches are obtained similarly but with different corresponding initial excitations.

The bifurcation diagram shown in Figure 2a is in good qualitative agreement with the results in [11]. For the current just above the threshold, the stable fundamental ML is achieved and can hold when increasing the current until *I* = 510 mA; from then on, an unstable ML regime with gradual shrinking of the pulse peak power is observed, which finally transits to a harmonic ML regime with approximately twice higher repetition rate. On the contrary, when decreasing the current from *I* = 620 mA, the second harmonic ML is established first, but limited in the left by an abrupt jump to the fundamental branch at *I* = 425 mA, indicating that the state with a pair of equal-spaced pulses propagating in the cavity is no longer a solution for this ML system at such low current. Similar transitions, at *I* = 460 mA in the third harmonic ML branch and at *I* = 425 mA and *I* = 510 mA in the fourth harmonic ML branch, are observed. This diagram is a simple way to identify different ML regimes and their robustness by noticing to which current this regime is still sustained. It is interesting to notice that a higher order harmonic ML always tends to relax to the closest and integral half-lower-order ML state, for example, the fourth harmonic ML jumps to the second harmonic branch first and then to the fundamental branch, omitting the third harmonic branch.

The numerical results here are quantitatively consistent with the experimental results in [3] in the terms of the average power (see Figure 2c), for example, the average power is about 11 mW at the current *I* that *I* − *I*_th_ = 150 mA. In addition, for higher gain currents, both QD ML lasers show multiple co-existing pulses during one cavity round trip time. However, the discrepancies in the peak power and pulse width values are mainly caused by the difference in the gain spectrum bandwidth (see Figure two (b) in [3]), which leads to higher peak power and shorter pulses in this work. Besides, in Figure 2, we can see that the threshold current (380 mA) is two times higher than that in [3]; this is due to the unidirectional propagation assumption in the DDE model which results to two times longer device length, and so consequently, for a similar threshold current density, the threshold current is doubled in this work.

**Figure 3:**
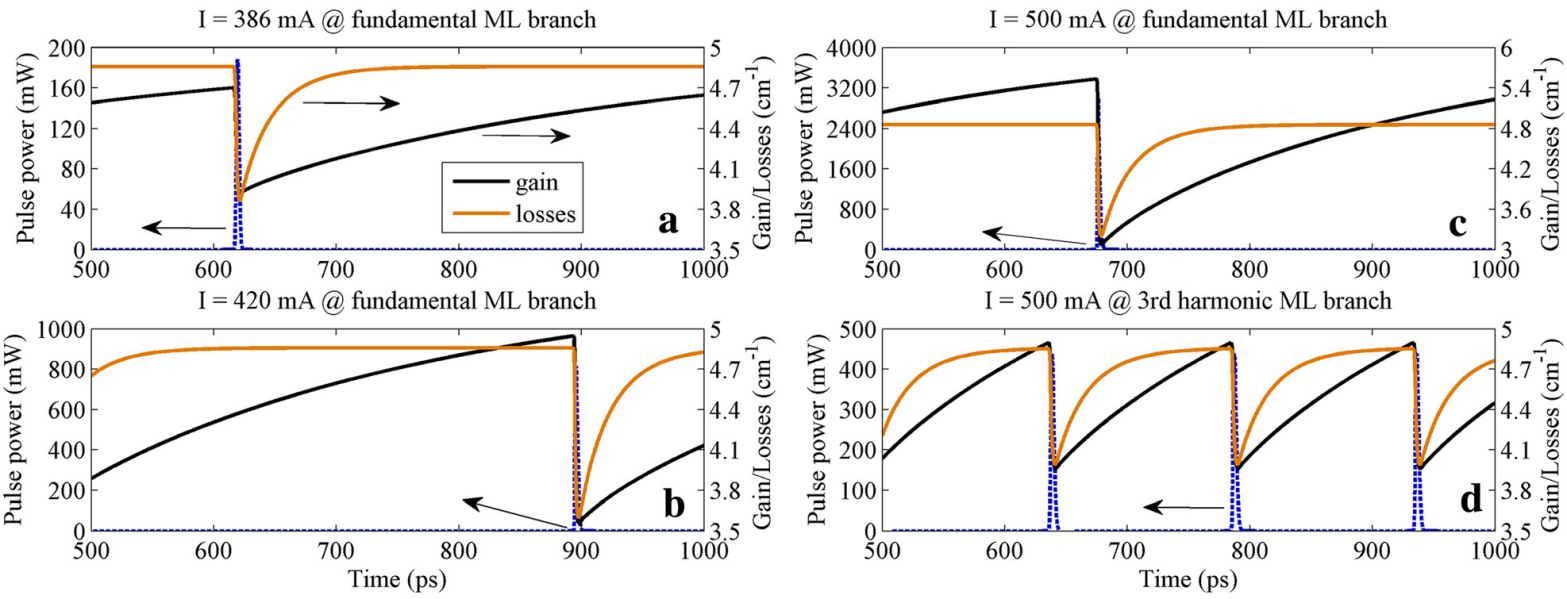
**The net gain window for the GS pulses at different current values indicated in Figure** 2 **a. (a)** *I* = 386 mA, **(b)** *I* = 420 mA, and **(c)** *I* = 500 mA in the fundamental ML branch and **(d)** *I* = 500 mA in the third harmonic ML branch. In the net gain window, the overall losses (brown line) and overall amplification (black line) experienced by the pulse during a complete round trip in the cavity are shown with the corresponding pulse envelope (blue dashed line). The pulse envelope in this figure corresponds to the optical intensity of the pulse propagating inside the device cavity, which relates with but is not equal to the output optical power.

The possibility of the existence of multi-pulse in the laser cavity can be attributed mainly to the relatively long round trip time of this long monolithic laser. The integrated losses *A*(*τ*) and gains *G*(*τ*) experienced by the pulse over a complete round trip within the cavity are presented in Figure 3, which shows the so-called net gain window (for a detailed explanation of this kind of figures, please refer to [12]). We can see that the high intensity of the optical pulse leads to gain/absorption depletion in the gain/SA section when the pulse comes, and then the gain/absorption would take a certain time to recover to the unsaturated initial value when the pulse is gone. Typically, the recovery time for the absorption is much faster, i.e., few tens of picoseconds, and can be significantly shortened by the applied reverse voltage down to 1 ps or less. Contrary to the absorption, the gain full recovery is found to occur in a few nanoseconds; what makes it even more special is that increasing the injection current only makes the gain recovery curve sharper but does not lead to noticeable reduction in the full recovery time (see Figure three in [12]).

For the device studied in this paper, the fundamental ML has a repetition period of about 460 ps. Therefore, at a current just above the lasing threshold (Figure 3a), fundamental ML is the only solution that satisfies the gain and absorption dynamics. We can see that the gain recovery rate is relatively slow now, so the gain is still smaller than the unsaturated losses before the following pulse comes, which helps to inhibit the background noise in the output. With the increase of the injection current, faster gain recovery and higher unsaturated gain are achieved; due to the long round trip period, even if it still cannot fully recover, the gain overcomes the unsaturated losses and establishes a positive net gain range before the arrival of the subsequent pulse (Figure 3b). Under this condition, fundamental ML can still be held to a certain extent. With further increase of the injection current, the positive net gain range will become even larger (Figure 3c) and finally results in the appearance of an additional pulse between each pair of original pulses by amplifying the background optical noise gradually, so the second harmonic ML appears. Since the absorption recovery time remains constant when increasing the current, the larger the current is, the earlier the gain exceeds the losses, and this condition makes the higher order harmonic ML become a possible solution of this ML system at a higher current (Figure 3d).

In our simulation, the spontaneous emission noise is highly reduced, leading to high inhibition of the spontaneous start-up of the harmonic ML. Therefore, the fundamental solution sustains up to a very high current level during the forward current sweeping. In the contrary, for the backward current sweepings, initial excitations with multiple pulses co-existing in the cavity are exploited, so the harmonic ML appears from the very beginning of the sweeping and holds until a current level where it is no longer a solution for the considered device. While in real experiments, due to the unavoidable spontaneous emission, automatic jump to harmonic ML at the onset current of the multistable regime should be expected when forward sweeps the injection current. In this study, we tried also the backward sweeping using initial excitations with five or even more co-existing pulses in one round trip time. In these kinds of situations, fast relaxation to the fourth harmonic ML is observed when numerical convergence was achieved. This fact indicates that, within the investigated current range, the relative relationship between the round trip time and the recovery times of gain and absorption could support, to the largest, four pulses co-existing in the laser cavity.

### Peak power improvement of the monolithic long-cavity ML laser

Now we focus on the fundamental ML regime. In some applications such as the two-photon microscopy, a pulse train with high pulse power and very low repetition rate is required. However, as demonstrated above, the maximum achievable peak power at the fundamental ML state is strictly limited by the early onset of the harmonic ML for lasers with repetition frequency from hundreds of MHz to few GHz. If this onset current is pushed to a higher value above the threshold current, obviously, higher peak power will be obtained.

**Figure 4:**
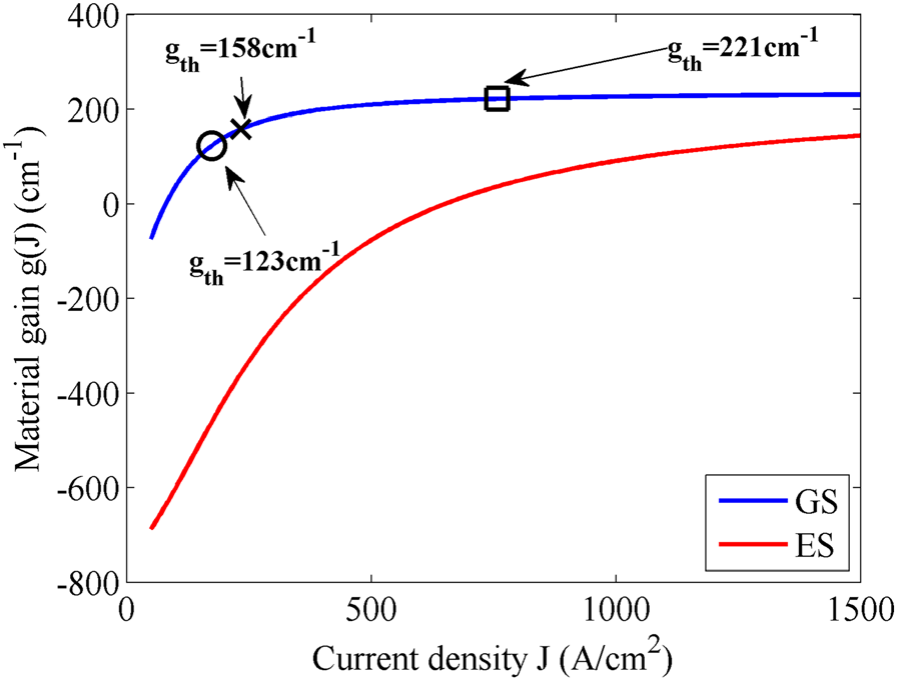
**QD material gain as a function of the injection current density for the considered device.** The material gains for the GS (blue line) and the ES (red line) are shown. The inserted first two markers indicate the threshold gain positions of the lasers without passive section (circle marker) and with a 4-mm-long passive section (cross marker). The last square marker corresponds to a threshold gain position which will be discussed later.

The GS and ES material gains *g*_*i*_/*Γ*_*xy*_ (*Γ*_*xy*_ = *Γ*_x_*Γ_*y*_) as a function of the injection current of the previously described device are shown in Figure 4. Based on the above discussions, the onset of the harmonic ML at high current could be partially attributed to the increase of the unsaturated gain which makes the gain overcome the losses easier. Since low repetition rate is our main target, the round trip time cannot be reduced; therefore, the only way to sustain the fundamental ML over a larger current span is to operate the laser in a condition of reduced differential gain (see Figure 4). Thanks to the reduced density of states, the QD medium always achieves early gain saturation at smaller current density compared with its bulk and quantum well counterparts, which means that the differential gain of this medium decreases rapidly when increasing the injection current, as shown in Figure 4. To achieve these operation conditions, we should increase the required threshold current of the laser, so that it would work at a current range with smaller differential gain. Different approaches have been attempted to obtain these favorable operation conditions as will be shown in the following.

The GS threshold gain of the considered devices has been estimated using the following approximate resonance equation at threshold:

3 $$ \frac{L_{\mathrm{g}}}{L}{\varGamma}_x{\varGamma}_y{g}_{\mathrm{th}}=\frac{L_{\mathrm{SA}}}{L}{\varGamma}_x{\varGamma}_y{\alpha}_{\mathrm{SA}\_0}+{\alpha}_i+\frac{1}{L} \ln \left(\frac{1}{\sqrt{R_0{R}_L}}\right) $$

where *L* = 2 cm is the device total length, *а*_SA_0_ = 585 cm^−1^ is the QD unsaturated absorption at the GS transition, and *а*_*i*_ = 2 cm^−1^ is the intrinsic waveguide losses. The last term on the right-hand side of the above equation represents the mirror losses *а*_m_ of the laser cavity. The calculated threshold gain of the previously analyzed device is *g*_th_ = 123 cm^−1^, while *g*_th_ = 158 cm^−1^ for the four devices whose results are discussed below (see Figure 4).

**Figure 5:**
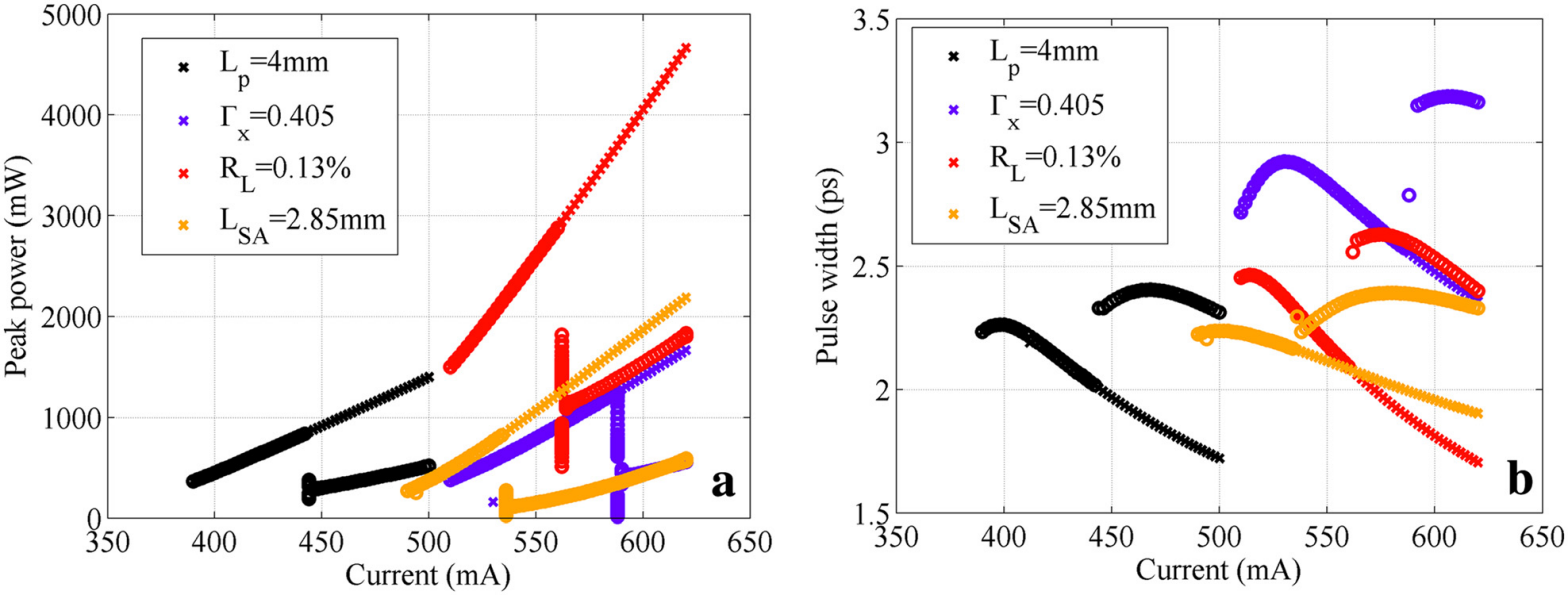
**Bifurcation diagrams of the peak power (a) and the pulse width (b) for the four modified devices.** These four devices are *L*_*p*_ = 4 mm (black), *Γ*_*x*_ = 0.405 (violet), *R*_*L*_ = 0.13% (red), and *L*_SA_ = 2.85 mm (yellow). For the sake of simplification, only the fundamental ML and the second harmonic ML branches are shown.

Four new 2-cm devices have been considered. For each of them, we changed only one structural parameter to push the threshold gain to 158 cm^−1^. The considered changes are as follows: forming a 4-mm passive section (*L*_g_ = 14 mm), reducing the ridge width so *Γ*_*x*_ = 0.405, applying anti-reflection coating so *R*_*L*_ = 0.13%, or increasing the SA length so *L*_SA_ = 2.85 mm (*L*_g_ = 17.15 mm). The peak power and pulse width bifurcation diagrams of these four devices are reported in Figure 5.

The original device could achieve a maximum peak power of about 650 mW before the onset current *I*_onset_ of the multistable regime (Figure 2a). According to the numerical results in Figure 5, these four modified devices all obtain higher output peak power before *I*_onset_, which verifies our theoretical prediction for improving the maximum achievable fundamental ML peak power. However, comparing the four devices, it is obvious that reducing the reflectivity at the output facet is the most efficient method, which results in the highest peak power and practically similar pulse width. Unlike other approaches, increasing mirror losses (*R*_*L*_ = 0.13%) not only moves device working point to the low differential gain region, but also preserves as much as possible the generated optical power and transfers it into the output. On the other hand, other approaches obtain higher threshold gain by introducing real optical attenuations or limiting the effective gain, hence sacrificing the optical power generated in the laser cavity.

We tried also to increase *g*_th_ up until 221 cm^−1^ where the gain is almost totally saturated (see Figure 4) and the differential gain is significantly reduced. However, at that condition, the threshold current is too high and overlaps with the harmonic ML onset current. Therefore, for achieving high peak power in fundamental pulse mode, the *g*_th_ is not always the higher the better; we should keep it away from the total saturation region in the gain curve.

## Conclusions

A modified multisection DDE model has been developed for the simulation of two-section or three-section ultra-long monolithic QD ML lasers. The multistable dynamical regimes of a 2-cm-long monolithic passively ML laser were studied. When changing the injection current, stable fundamental ML and harmonic ML up to the fourth order have been observed. According to our analysis, the possibility of a multistable ML regime appearing should be attributed to the relatively shorter gain recovery time at high current compared with the repetition period and the fast increase of the gain when increasing the injection current. Therefore, in order to obtain higher fundamental ML peak power, four modified lasers with higher threshold gains were considered. The numerical results validated our theoretic analysis that device working at a regime with smaller differential gain could push the onset of harmonic ML to a higher current level. In addition, we have also observed that reducing the output reflectivity is the most efficient way to reach higher fundamental ML peak power, since this method increases the cavity losses of the optical power by transferring them into the output power.
